# CAN THE BRIEF INTERVIEW OF MENTAL STATUS (BIMS) IDENTIFY RISK FOR IMPAIRED IADLS DUE TO FUNCTIONAL COGNITIVE DEFICITS

**DOI:** 10.1093/geroni/igz038.1891

**Published:** 2019-11-08

**Authors:** Timothy Marks, Dorothy F Edwards, Muhammad O Al- heizan, Gordon M Giles

**Affiliations:** 1 University of Wisconsin Madison, Madison Wisconsin, Wisconsin, United States; 2 University of Wisconsin Madison, Madison, Wisconsin, United States; 3 Samuel Merritt University, Oakland, California, United States

## Abstract

The Brief Interview of Mental Status (BIMS) was introduced to the Minimum Data Set (MDS) 3.0 as a cognitive screening tool. It includes temporal orientation and word recall (Saliba et al., 2012). This study examined the ability of the BIMS to identify impairment on performance-based functional cognitive screening tests that assess instrumental activities of daily living (IADLs). We recruited a cross-sectional sample of 200 participants who met the following inclusion criteria: age 55 and older, living independently in the community, and able to read and communicate in English.. Participants ranged in age from 55 to 92 years (Mean 70.96:SD = 8.56),were predominantly White (68%) and female (65%). Participants were administered the BIMS and a battery of performance-based tests of functional cognition -the Performance Assessment of Self Care Skills and the Weekly Calendar Planning Activity (WCPA). There was a mismatch in screening results: Among Individuals identified as cognitively intact on the BIMS 22-45% were found to be impaired on the PASS and/or the WCPA. Sensitivity of the BIMS to identify impaired IADL function did not exceed .06, although specificities were high (< .95). These findings suggest that individuals categorized as cognitively normal by the BIMS may be impaired on more complex IADL tasks. Individuals classified as unimpaired on the BIMS, may benefit from more complex functional cognitive screening to further assess IADLs function to better estimate ability to live independently in the community. Performance based assessments can improve discharge planning by identifying elders at risk after hospitalization.

